# Human Platelet Protein Ubiquitylation and Changes following GPVI Activation

**DOI:** 10.1055/s-0038-1676344

**Published:** 2018-12-31

**Authors:** Amanda J. Unsworth, Izabela Bombik, Adan Pinto-Fernandez, Joanna F. McGouran, Rebecca Konietzny, René P. Zahedi, Steve P. Watson, Benedikt M. Kessler, Catherine J. Pears

**Affiliations:** 1 Department of Biochemistry, University of Oxford, Oxford, United Kingdom; 2 Institute for Cardiovascular and Metabolic Research, University of Reading, Reading, United Kingdom; 3 Nuffield Department of Medicine, Target Discovery Institute, University of Oxford, Oxford, United Kingdom; 4 JGH Proteomics Centre, Lady Davis Institute, Jewish General Hospital, Montreal, Quebec, Canada; 5 Institute of Cardiovascular Sciences, College of Medical and Dental Sciences, University of Birmingham, Edgbaston, Birmingham, United Kingdom; 6 Centre of Membrane Proteins and Receptors, Universities of Birmingham and Nottingham, The Midlands, United Kingdom

**Keywords:** platelets, sites of ubiquitinylation, GPVI, deubiquitylation inhibition

## Abstract

Platelet activators stimulate post-translational modification of signalling proteins to change their activity or their molecular interactions leading to signal propagation. One covalent modification is attachment of the small protein ubiquitin to lysine residues in target proteins. Modification by ubiquitin can either target proteins for degradation by the proteasome or act as a scaffold for other proteins. Pharmacological inhibition of deubiquitylases or the proteasome inhibition of platelet activation by collagen, demonstrating a role for ubiquitylation, but relatively few substrates for ubiquitin have been identified and the molecular basis of inhibition is not established. Here, we report the ubiquitome of human platelets and changes in ubiquitylated proteins following stimulation by collagen-related peptide (CRP-XL). Using platelets from six individuals over three independent experiments, we identified 1,634 ubiquitylated peptides derived from 691 proteins, revealing extensive ubiquitylation in resting platelets. Note that 925 of these peptides show an increase of more than twofold following stimulation with CRP-XL. Multiple sites of ubiquitylation were identified on several proteins including Syk, filamin and integrin heterodimer sub-units. This work reveals extensive protein ubiquitylation during activation of human platelets and opens the possibility of novel therapeutic interventions targeting the ubiquitin machinery.

## Introduction

Platelets play a primary role in haemostasis following vascular damage. The major mediators of platelet responses interact with receptors which converge on activation of the serine/threonine kinase protein kinase C and increases in intracellular Ca ^2+^ levels. Soluble mediators such as thrombin signal through receptors coupled to heterotrimeric G proteins, whereas the glycoproteins (GPs) GPVI and CLEC-2 signal through Src family kinases (SFKs) and Syk. 1 2 3 All pathways lead to covalent modification of target proteins to cause changes in activity or association with other cellular components. The best studied of these modifications is phosphorylation, but it is apparent that other modifications play an important role in modulating platelet responses.

One modification of increasing interest is protein ubiquitylation which is vital to a wide range of cellular responses. 4 Ubiquitin is a 76 amino acid residue protein that is ligated to lysine residues (K) in target proteins. Ubiquitin itself contains seven Ks to which further ubiquitin molecules can be ligated to generate a diversity of polyubiquitin chains with different structures, as well as linear polymers of ubiquitin. 5 Modification is a three-step reaction. E1-mediated activation of ubiquitin is followed by conjugation of ubiquitin to an E2 enzyme. This is then targeted directly or indirectly via an E3 ligase to a target substrate. A variety of E1, E2 and E3 enzymes have been described as well as deubiquitylating enzymes. Ubiquitin chains are recognized by proteins with ubiquitin binding domains that are specific for particular linkages. These proteins can target the modified protein for degradation by the proteasome or lead to complex formation to propagate a signalling response. Classically, K48-polyubiquitin targets proteins to the proteasome for degradation. 6 Other linkages such as K63-linked ubiquitin chains and mono-ubiquitin are important in signalling and receptor trafficking. 7

A role for the proteasome in platelet functional responses has been suggested as the proteasome inhibitor bortezomib, used in the clinic for the treatment of multiple myeloma and mantle cell lymphoma, inhibits platelet activation by thrombin, adenosine diphosphate (ADP) and collagen. 8 9 10 Proteasome inhibitors block thrombus formation in a mouse FeCl _3_ model. Pre-treatment of platelets with inhibitors of deubiquitylases also block platelet aggregation in response to these agonists. 11 In addition, there is evidence that ubiquitin is involved in signalling via production of scaffolds. The tyrosine kinase Syk is ubiquitylated on activation of GPVI in human platelets. 12 13 Ubiquitylation requires activity of SFKs, and ubiquitylated Syk has increased kinase activity. 12 The ubiquitin ligase c-Cbl binds to Syk phosphorylated on Tyr317. 14 Mice deficient in c-Cbl do not show ubiquitylation of Syk, consistent with c-Cbl being the responsible E3 ligase. Platelets from c-Cbl-deficient mice show hyper-phosphorylation of signalling proteins and increased responses to GPVI agonists suggesting that ubiquitin modification promotes down-regulation of Syk and ubiquitylation plays an important role in platelet functional responses. 12 13 It has been proposed that ubiquitylation of Syk promotes binding of the tyrosine phosphatase TULA-2 leading to down-regulation of signalling by Syk. Loss of TULA-2 promotes Syk hyper-phosphorylation and platelet hyperactivation. 15 16 17

Proteomic approaches have been applied to human platelets to characterize the complete proteome as well as sub-sets of proteins that are secreted, phosphorylated or shed (reviewed in Refs. 18 ^,^ 19 ^,^ and 20 21 22 ). Complete proteome analysis identified several other proteins involved in ubiquitin metabolism in human platelets including E1, E2, E3 ubiquitin ligases, deubiquitylases and proteins with ubiquitin binding domains, suggesting a more general role for ubiquitin in platelet biology. 21 For example, the E3 ligase RNF181 associates with the intracellular domain of the major platelet integrin αIIbβ3. 23 Further, several other proteins have been shown to be ubiquitylated in platelets including filamin and talin. 10

In this study, we have mapped sites of ubiquitylation of proteins in resting platelets and following stimulation of GPVI with collagen-related peptide (CRP-XL). Cleavage of proteins with trypsin leaves a characteristic tag of two glycine residues ligated to lysines modified by ubiquitylation. Antibodies directed against this diGly-tag were used to enrich for tagged peptides. Mass spectrometry (MS) reveals 1,634 peptides containing diGly-tags derived from 691 proteins. Of these, 1,116 tagged peptides from 476 proteins were detected in resting platelets, consistent with widespread protein ubiquitylation. Importantly, marked changes in the ubiquitylation state (both increases and decreases) are seen in response to GPVI activation. Note that 905 diGly-tagged peptides showed a change of more than twofold, including numerous sites in Syk, talin and filamin, as well as in other proteins known to be involved in platelet functional responses. Our analysis points to a general role for ubiquitylation in platelet regulation. An understanding of the platelet ubiquitome and its changes will identify novel targets for therapeutic intervention.

## Materials and Methods

### Materials

FK2 antibody was purchased from Enzo Life Sciences; antibodies against actin, PLCγ2 and Syk (N19 and 4D10) from Santa Cruz; LAT, SLP-76 from Millipore; PKCδ and PE-Cy5 anti-CD62P from BD Biosciences; phospho-specific antibodies for Syk (Y525/526, Y348, Y323) from Cell Signaling; and fluorescein isothiocyanate (FITC)-conjugated anti-fibrinogen antibody was from Agilent Technologies. PR619 and GST-Tandem Ubiquitin Binding Entities (GST-TUBEs) were from Tebu-Bio Ltd, and Protein A-Sepharose (PAS) and NuPage gradient gels from Invitrogen. Other reagents were from Sigma (Poole, United Kingdom).

### Preparation of Human Washed Platelets

Studies on human platelets were performed with ethical approval from the Oxfordshire Research Ethics Committee (Ref:08/HO605/123). Blood was drawn from aspirin-free, healthy consenting volunteers. Whole blood was drawn into one-tenth total volume sodium citrate and twice-washed platelets were prepared as previously described. 13 For the proteomic analysis, an additional centrifugation step was introduced to remove leukocytes; platelet-rich plasma was diluted in 1:1 ratio with modified Tyrode's Hepes buffer (138 mM NaCl, 2.7 mM KCl, 1 mM MgCl _2_ , 3 mM NaH _2_ PO _4_ , 5 mM glucose, 10 mM HEPES), pH 7.3 and centrifuged at 240 × *g* for 10 minutes as described. 21 Pelleted platelets were re-suspended in modified Tyrode's Hepes buffer, containing 0.2 U/mL apyrase and 10 µM indomethacin, adjusted to 5 × 10 ^8^ platelets/mL (for immunoprecipitation and blotting) or 1.5 × 10 ^9^ platelets/mL (for proteomics) and left to rest for 1 hour at 37°C. Platelets were pre-incubated for 5 minutes in the presence of dimethyl sulfoxide (0.1% v/v), PR619 or MG132. Aggregation and dense granule secretion were monitored as previously described. 24

### Fluorescence-Activated Cell Sorting Measurement of Fibrinogen Binding and P-Selectin Exposure

Measurements of fibrinogen binding and P-selectin exposure were performed using washed platelets pre-treated with or without PR619 (10 µM) and stimulated with CRP-XL (3 µg/mL) in the presence of FITC-conjugated polyclonal rabbit anti-fibrinogen antibody and PE-Cy5-conjugated mouse anti-CD62P antibody, and then incubated for 20 minutes in the dark. Platelets were then fixed by addition of filtered formyl saline (0.2% formaldehyde in 0.15 M NaCl) and median fluorescence intensities were measured for 5,000 events in the platelet gate (determined by forward and side scatter profiles) per sample on an Accuri C6 Flow Cytometer (BD Biosciences, United Kingdom) using the CFlow Sampler software.

### DiGly Pull-Down for Mass Spectrometry

Platelets were prepared from 300 mL of blood from three donors (100 mL per donor) for each experiment and left untreated or stimulated with CRP-XL (10 μg/mL) for 5 minutes at 37°C. A total of six different donors were used over the three experiments: (Experiment 1: Donors A, B, C: Experiment 2: Donors C, D, E: Experiment 3: Donors A, D, F). Reactions were stopped by addition of 2× NP40 lysis buffer (0.5% NP40, 50 mM Tris, pH 7.4, 150 mM NaCl, 20 mM MgCl _2_ , 2 mM N-ethylmaleimide). Lysates were used for GlyGly immunoprecipitation using PTMScan Ubiquitin Remnant Motif Kit (Cell Signaling), according to manufacturer's protocol. Briefly, extracts were solubilized and denatured in 10 mL lysis buffer (20 mM HEPES, pH 8.0, 9 M urea, 1 mM sodium orthovanadate, 2.5 mM sodium pyrophosphate, 1 mM β-glycerophosphate), reduced using dithiothreitol (4.5 mM final) for 30 minutes at 55°C. These are conditions known to cause carbamylation but this will not influence the existing covalent diGly modifications and the mass of carbamylation (+43 Da) is distinguishable from the diGly adduct (+114.1 Da). This was followed by alkylation using iodoacetamide (100 mM final) for 15 minutes at room temperature in the dark. Possible artefacts due to over-alkylation by iodoacetamide, which can be misinterpreted as a diGly modification, 25 are not observed when the alkylation step is performed at room temperature. 26 27 Samples were subsequently diluted fourfold in 20 mM HEPES, pH 8.0 (∼2 M urea final), followed by digestion with trypsin-TPCK (Worthington, LS003744, 10 mg/mL final) overnight at 37°C. Samples were then acidified using trifluoroacetic acid (1% final), and desalted using C-18 Sep-Pak (Waters) cartridges according to the manufacturer's protocol. Peptides were lyophilized and re-suspended in 1.4 mL immunoprecipitation IAP buffer (PTMScan), and the remaining insoluble material cleared by centrifugation and anti-GlyGly antibody beads added followed by rotation and kept 4°C overnight. Beads were subsequently washed twice using 1 mL IAP buffer, followed by three water washes. Immunoprecipitated material was eluted twice in 55 and 50 μL 0.15% trifluoroacetic acid in water. Eluates were pooled and precipitated using chloroform and methanol. 28 Peptide material was re-suspended in 20 μL water with 0.1% trifluoroacetic acid, followed by stage-tip desalting and concentration. 29 Peptide eluate material was dried by vacuum centrifugation, and stored at –80°C.

### Mass Spectrometry and Data Analysis

Immunoprecipitated peptide material was re-suspended in 20 μL water with 0.1% trifluoroacetic acid and 2% acetonitrile. Samples were injected into a nano-UPLC U3000 system (ThermoFisher) coupled to an Orbitrap Fusion Lumos tandem mass spectrometer (MS/MS) (ThermoFisher) as described previously. 30 Peptide material from TUBE pull-down experiments was analysed using a Waters nano-UPLC Acquity system coupled to an Orbitrap Velos mass spectrometer (ThermoFisher) as described previously. 31

MS raw data were processed using the ProteoWizard software (Msconvert) and subsequently analysed using the Mascot (Matrixscience) search engine (v2.5.1) using the UniProt human protein databases (retrieved June 2011 and October 2014) for searches. Alternatively, for label-free quantitative analysis, MS raw data were processed using Progenesis QI for proteomics (QIP) software (v3.0; Nonlinear Dynamics, Newcastle-upon-Tyne, United Kingdom). MS/MS spectra were searched against the UniProt human sequences (retrieved October 2014) using Mascot. Precursor mass tolerance was set to 10 ppm and the fragment ion tolerance to 0.05 Da. Deamidation on asparagine and glutamine, diGly adducts on lysine/cysteine, carbamidomethylation on cysteine and oxidation on methionine residues were included as variable modifications. Peptide false discovery rate (FDR) was set to 1%, and all peptides with an ion score of greater than 20 were imported into Progenesis QIP. Proteins that were defined with at least one unique peptide were included in the protein dataset for further analysis. For normalization, protein abundance values were centred on the median abundance of the 90% of proteins with the lowest variance across all runs. Processed data were analysed and visualized using the Venny 2.1 and Perseus software tools (v1.6.0.2). For the analysis by volcano plot, we used Student's *t* -test for the comparison of stimulated versus unstimulated conditions, which was corrected for multiple testing using permutation FDR as previously described. 32 The MS data have been deposited to the ProteomeXchange Consortium via the PRIDE partner repository with the dataset identifier PXD009072 and 10.6019/PXD009072. 33

### Immunoprecipitation and Pull-Down of Ubiquitylated Protein

Reactions were stopped by addition of an equal volume of cold NP40 lysis buffer (150 mM NaCl, 25 mM Tris [pH 7.6], 1% NP-40, 2 mM ethylenediaminetetraacetic acid, 1 mM ethylene glycol tetraacetic acid, 1 mM sodium orthovanadate, 100 μM 4-(2-aminoethyl)-benzenesulfonyl fluoride, 1 μg/mL pepstatin and 10 μg/mL leupeptin). Following incubation on ice for 15 minutes to ensure lysis, lysates were pre-cleared using PAS beads. Proteins were immunoprecipitated overnight at 4°C with 5 μg of appropriate antibodies plus PAS (25 μL/500 μL lysate). Syk was immunoprecipitated using BR15 (kind gift of M. Tomlinson, DNAX Research Institute, Palo Alto, California, United States). Immunoprecipitates were washed five times with lysis buffer and beads boiled in 2× sodium dodecyl sulphate Laemmli buffer. GST-TUBEs 34 were used as per the manufacturer's instructions. Briefly, GST-TUBEs were added to platelet lysates for 15 minutes on ice. Lysates were then clarified by centrifugation at 10,000 × g for 10 minutes, before being subjected to standard to glutathione agarose affinity purification. Bound proteins were eluted using reduced glutathione.

## Results

### Ubiquitylation of Platelet Proteins

**Fig. 1:**
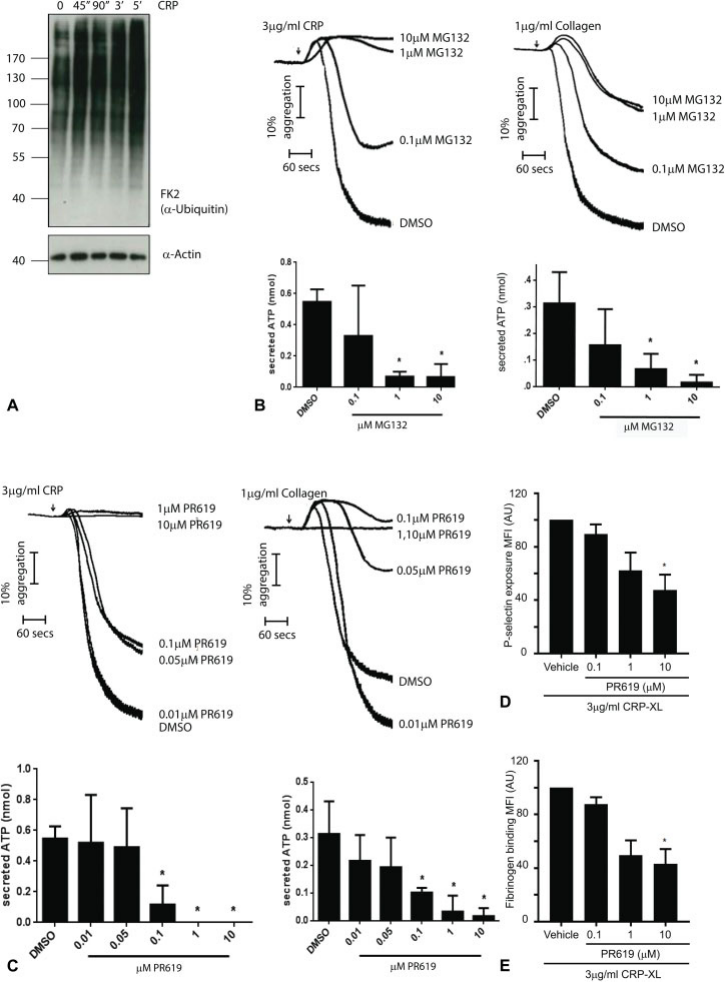
Protein ubiquitylation in resting human platelets and on activation of glycoprotein (GP) VI. ( **A** ) Human platelets were allowed to rest before stimulation with 3 μg/mL collagen-related peptide (CRP-XL) for the times shown. Whole cell lysates were resolved by sodium dodecyl sulphate-polyacrylamide gel electrophoresis (SDS-PAGE) and blotted using FK2 antibody that recognizes both mono- and polyubiquitin. The samples were re-probed with actin to control for protein loading. ( **B, C** ) Human platelets were stimulated with CRP-XL (3 μg/mL) or collagen (1 μg/mL) in the presence of increasing concentrations of proteasome inhibitor MG132 (B) or pan deubiquitylase inhibitor PR619 (C). Aggregation and dense granule secretion were monitored as previously described. 24 ( **D, E** ) Alpha granule secretion was measured by detecting levels of P-selectin exposure (D) and activation of the integrin αIIbβ3 by detecting levels of fibrinogen binding (E) at the platelet surface by flow cytometry using PE/Cy5 anti-human CD62P and fluorescein isothiocyanate (FITC)-labelled anti-fibrinogen antibody, respectively, in washed platelets treated with 3 μg/mL CRP-XL. Using a BD Accuri C6 flow cytometer, 5,000 events were analysed using the CFlow Sampler software.

Western blot using an antibody (FK2) that recognizes both mono- and poly-ubiquitin revealed the ubiquitylation of multiple proteins in resting human platelets ( Fig. 1A ). The absence of prominent bands is consistent with ubiquitylation of many proteins and/or a range of ubiquitylation states. The level of total protein ubiquitylation appeared to increase only modestly on stimulation by CRP-XL. However, consistent with previous reports using collagen, 8 CRP-XL-dependent platelet aggregation and dense granule secretion are inhibited in a dose-dependent manner by proteasome inhibitors MG132 ( Fig. 1B ) and bortezomib (data not shown) suggesting an important role for ubiquitylation and the proteasome downstream of GPVI. In addition, CRP-XL-induced aggregation and secretion of both dense and α granules are inhibited by the pan-deubiquitylase inhibitor PR619 ( Fig. 1C , D ), consistent with previous reports using collagen. 11 PR619 also inhibits CRP-XL-induced activation of the fibrinogen binding by the major platelet integrin αIIbβ3 ( Fig. 1E ), consistent with inhibition of the GPVI signalling pathway upstream of integrin activation. Inhibition of platelet activation by low concentrations of both proteasome and deubiquitylase inhibitors, at concentrations which do not inhibit platelet activation by other agonists such as thrombin 8 10 11 (and data not shown), suggests a dependence on removal of ubiquitin from proteins for CRP-XL-induced platelet activation.

**Fig. 2:**
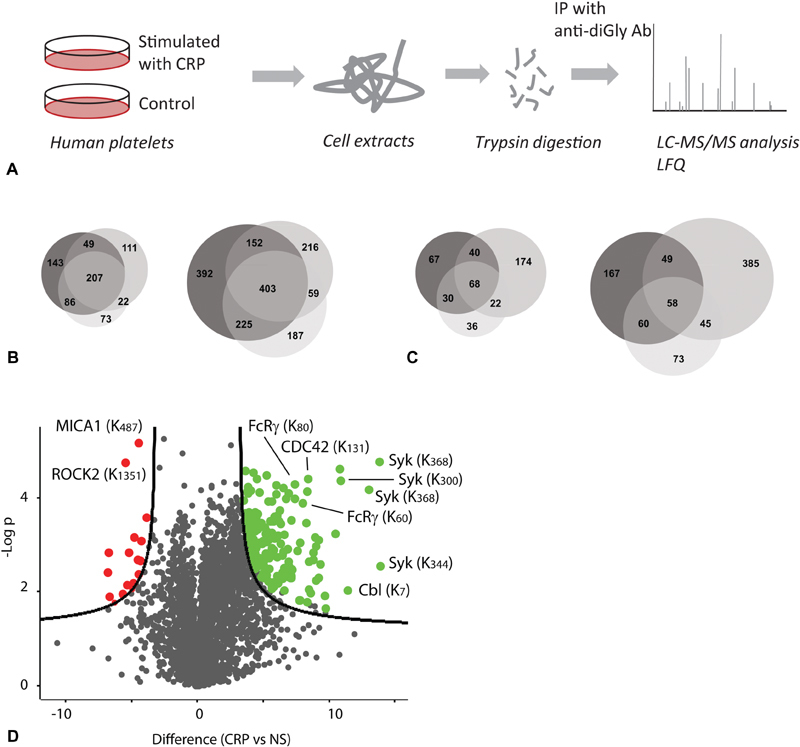
Analysis of diGly-tagged peptides detected by mass spectrometry in resting human platelets and changes on stimulation with collagen-related peptide (CRP-XL). ( **A** ) Schematic of workflow used to identify diGly-tagged peptides from human platelets. Cells were collected, lysed and lysates reduced and alkylated prior to digestion with trypsin. Alkylation was performed using iodoacetamide at room temperature in the dark to exclude possible artefacts due to over-alkylation of lysine residues. 25 26 27 Gly-Gly peptides were enriched by immunoprecipitation followed by desalting, concentration and subjected to label-free quantitative analysis by liquid chromatography–tandem mass spectrometry (LC-MS/MS). ( **B** ) Venn diagrams showing the overlap of both proteins (left panel) and peptides (right panel) containing diGly tags on lysine residues as detected by mass spectrometry in three independent experiments. ( **C** ) Venn diagrams showing overlap of proteins (left panel) and peptides (right panel) containing diGly tags on lysine residues showing a significant change in abundance of at least twofold in three independent experiments. ( **D** ) Volcano plot representation of changes in abundance of diGly peptides following platelet activation, highlighting enhanced ubiquitylation of Syk, CDC42 kinase, FcRγ-chain and c-Cbl and reduced ubiquitylation of ROCK2.

To identify sites of ubiquitylation, proteins were extracted from control and CRP-XL (10 μg/mL) stimulated human platelets, and digested with trypsin to leave a characteristic diGly-tag on lysines to which ubiquitin has been conjugated ( Fig. 2A ). Peptides containing the diGly-tag were enriched by immunoprecipitation using an antibody specific for the tag and identified by MS ( Supplementary Table S1 , available in the online version). This analysis was performed three times, in each case pooling platelets from three individuals. In total, platelets from six individuals were included in the analysis, three of these donating on two occasions (see the “Methods” section for details).

Considering all peptides before and after CRP-XL stimulation revealed in total 1,634 peptides containing diGly-tags on lysine residues, derived from 691 proteins. Comparing the three repeats, 403 peptides from 207 proteins were detected in all three experiments and a further 436 peptides from 157 proteins in two repeats. Therefore, 51% of the peptides were identified more than once ( Fig. 2B ). This level of variation between experiments is consistent with previous reports for sites of ubiquitylation from tissue samples (rather than cell lines). A similar analysis of mouse liver tissue showed 72% of sites were present in at least two experiments. 35 Of note, these mice were inbred and living in a controlled environment minimizing variation between individuals. In a recent analysis of the phosphoproteome of human platelets, 50.6% of phosphopeptides identified were found in only one experiment out of three. 36 Sites identified in only one experiment could represent differences between individuals (inherent or environmental) or technical variation between experiments, but all are included in Supplementary Table S1 (available in the online version) for maximal coverage.

In resting platelets, 1,116 diGly-tagged peptides from 476 proteins were detected, some proteins containing a large number of sites including cytoskeletal proteins filamin (25 sites), Talin (53) and myosin-9 (25). There was ubiquitylation of several small G proteins (9 different rabs, rap1b, rac1 and 2, cdc42) and receptors associated with inhibitory signalling (platelet endothelial cell adhesion molecule-1, junctional adhesion molecule-A and endothelial cell–selective adhesion molecule-1). Sites were also apparent on some proteins involved in platelet activation such as three sites on the Fc receptor γ (FcRγ)-chain, which forms a complex with GPVI, and several proteins involved in Ca ^2+^ signalling such as P2X1 receptor (2 sites), Stim1 (7) and the Ca ^2+^ -transporting ATPase ATP2C1 (9).

### Changes in diGly Peptides following Stimulation of GPVI

Quantification of changes in diGly peptide abundance following CRP-XL stimulation (5 minutes) revealed sites that increased and decreased while others did not change. Supplementary Table S2 (available in the online version) shows all peptides from Supplementary Table S1 (available in the online version) where there is significant confidence ( *p* < 0.05) in abundance changes between technical replicates. Supplementary Table S3 (available in the online version) shows only those with changes of more than twofold following CRP-XL stimulation. The overlap seen between experiments was comparable to that seen for the total number of sites ( Fig. 2C ). Note that 716 diGly-tagged peptides showed a significant increase in abundance of more than twofold (18 from 11 different proteins were only seen in stimulated samples, 72 from 43 proteins increased more than 50-fold and 299 from 191 proteins more than 10-fold in at least one repeat). Peptides from components of the GPVI signalling pathway such Syk and the FcRγ-chain are among the proteins showing large increase in abundance with high confidence, as apparent on a volcano plot ( Fig. 2D ). Note that 445 peptides did not change by twofold in at least one repeat. Also, 209 peptides from 142 proteins reduced significantly by more than twofold (5 were not detected following stimulation), with 46 peptides from 36 proteins reduced by more than 10-fold, and 9 from 9 proteins by more than 50-fold in at least one repeat.

### Signalling Proteins

**Fig. 3:**
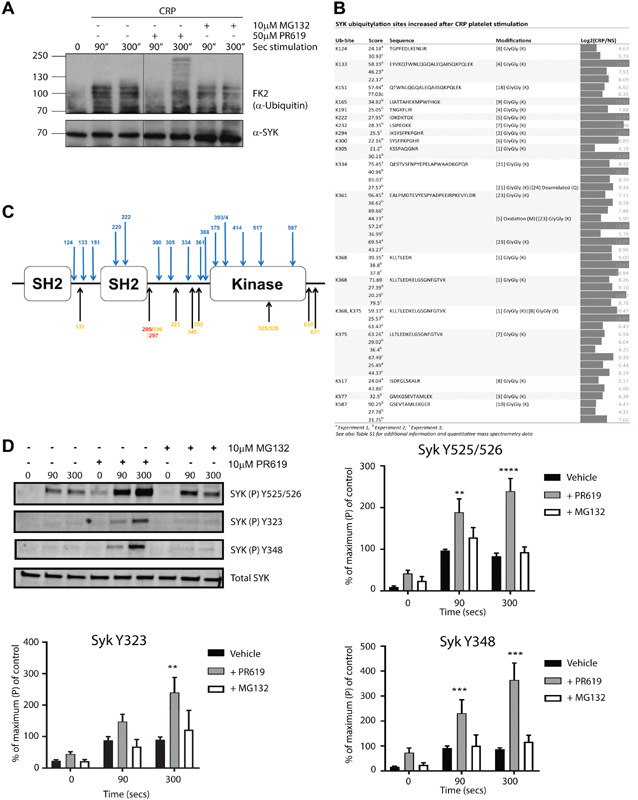
Ubiquitylation of Syk and phospholipase Cγ2 in human platelets following activation of glycoprotein (GP) VI receptor with collagen-related peptide (CRP-XL). ( **A** ) Human platelets were stimulated with CRP-XL (3 μg/mL) in the presence or absence of inhibitors of the proteasome inhibitor MG132 (10 μM) and the pan-deubiquitylase inhibitor PR619 (50 μM). Syk was immunoprecipitated at the times shown and the resulting Western blot probed with an antibody to ubiquitin (FK2). Samples were re-probed with anti-Syk as a loading control. ( **B** ) The site of the diGly-tag on lysine residues of peptides derived from Syk that were detected in human platelets are shown along with their fold stimulation in the three independent experiments. The peptide score as determined using Progenesis, the peptide sequence and site of modification are also shown. ( **C** ) Schematic diagram showing the location of the sites of ubiquitylation identified in the human Syk protein relative to its SH2 and kinase domains. Sites of ubiquitylation as detected as diGly-tagged lysine residues are shown in blue. Known major phosphorylation sites are shown in orange (tyrosine) and red (serine). ( **D** ) Human platelets were incubated for the times shown with vehicle control or 3 μg/mL CRP-XL following 10 minutes of pre-incubation with vehicle, 10 μM PR619 or 10 μM MG132. Whole cell lysates were analysed by Western blot using antisera specific for phosphorylated forms of Syk and total Syk. One representative experiment shown. Data from three independent experiments were quantified and the average, ± standard error of the mean (SEM) shown for each condition.

**Fig. 4:**
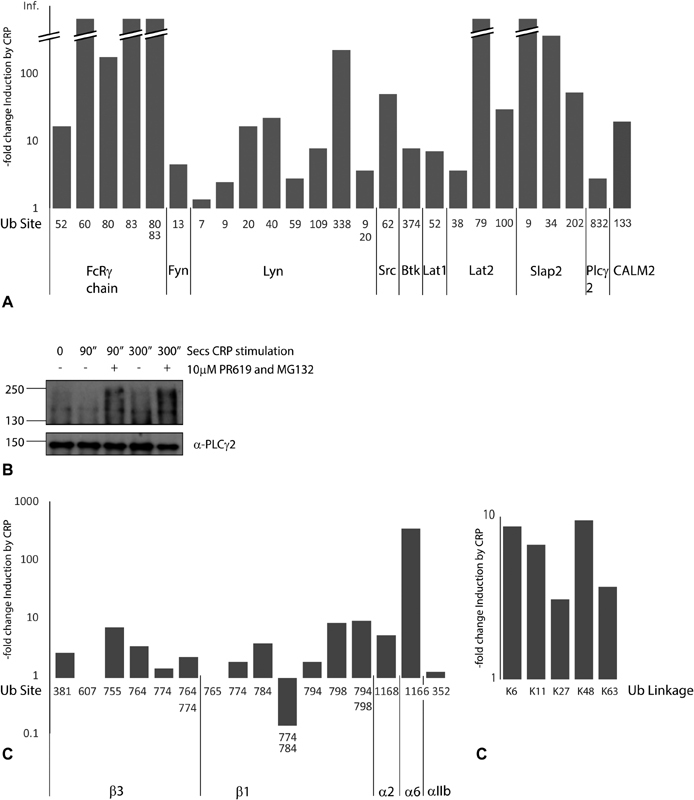
Ubiquitylation sites detected by mass spectrometry in human platelets. ( **A** ) Components of the glycoprotein (GP) VI signalling pathway. The site of the diGly-tag on lysine residues of peptides derived from components of the GPVI signalling pathway that were detected in human platelets are shown along with their maximum fold stimulation in the three independent experiments. ( **B** ) Human platelets were stimulated with collagen-related peptide (CRP-XL) (3 μg/mL) in the presence or absence of the proteasome inhibitor MG132 (10 μM) and the pan-deubiquitylase inhibitor PR619 (10 μM). PLCγ2 was immunoprecipitated at the times shown and the resulting Western blot probed with an antibody to ubiquitin (FK2). Samples were re-probed with anti-PLCγ2 as a loading control. ( **C** ) Integrin sub-units. The site of the diGly-tag on lysine residues of peptides derived from integrin sub-units that were detected in human platelets are shown along with their maximum fold stimulation in the three independent experiments. ( **D** ) Ubiquitin linkages. The site of the diGly-tag on lysine residues of peptides derived from ubiquitin that were detected in human platelets are shown along with their maximum fold stimulation in the three independent experiments. The peptide score, the peptide sequence and site of modification are also shown.

Several proteins from the GPVI signalling pathway contained diGly-tagged peptides. Immunoprecipitation of Syk followed by Western blot confirmed an increase in ubiquitylation on CRP-XL treatment ( Fig. 3A ), exacerbated by PR619. Syk has 17 ubiquitylation sites, all increasing on CRP-XL treatment ( Fig. 3B ), consistent with previous reports of extensive laddering of Syk on GPVI activation. 12 13 The sites are scattered throughout the protein including a cluster of three sites between K361 and K375 in the kinase domain of Syk ( Fig. 3C ). The sites showing the highest increases (K300, 334 and 368), also cluster in this central region. A peptide with two tags (K368, 375) revealed that individual molecules of Syk can be modified on adjacent sites. The increase in ubiquitylation of Syk observed in the presence of PR619 was associated with an increase in phosphorylation on several tyrosine residues, namely, Y525/526, Y323 and Y345 ( Fig. 3D ). Inhibition of the proteasome by MG132 did not lead to a detectable change in ubiquitylation ( Fig. 3A ) or a change in phosphorylation of these same sites ( Fig. 3D ) in Syk. A second tyrosine kinase, Lyn, shows seven ubiquitylation sites ( Fig. 4A ), all increasing on activation, with one peptide containing two modifications (K9, 20). The FcRγ-chain is ubiquitylated on four lysine residues, all increasing robustly on CRP-XL stimulation. One peptide containing two modifications (K80, 83) was detected in all three experiments. In contrast, a single site was detected for the tyrosine kinases Btk, Fyn and Src and phospholipase C (PLC)γ2 ( Fig. 4A ). Western blot of immunoprecipitates of PLCγ2 reveals ubiquitylation which increases on treatment with CRP-XL and is enhanced by the presence of MG132 and PR619 ( Fig. 4B ). The adaptor proteins LAT1, LAT2 and SLAP-2 all have sites which increase. Three sites in c-Cbl are up-regulated in CRP-XL-stimulated platelets, consistent with a role in signalling downstream of GPVI. In addition to the tyrosine kinase-dependent signalling pathway, the TRAF4 component of nicotinamide adenine dinucleotide phosphate oxidase complex also binds directly to the cytoplasmic tail of GPVI to regulate redox responses and calmodulin binds directly to the TRAF4 binding sequence. 37 TRAF4 was not identified in our analysis but three ubiquitylation sites were found on calmodulin, which also associates with the GPVI tail, one of which increased by 14-fold in one experiment ( Fig. 4A ; Supplementary Tables S1 , S2 and S3 , available in the online version).

Other proteins involved in platelet activation also showed increased ubiquitylation such as pleckstrin (seven sites). Receptors for other platelet agonists such as the protease-activated receptor 4 thrombin receptor and the purinergic receptor P2X1 are targets for ubiquitylation as are integrin sub-units ( Fig. 4C ). Three ubiquitylation sites in the C-terminal tail of the β3-integrin sub-unit were identified. Peptides containing two diGly-tags on adjacent lysines were apparent revealing simultaneous ubiquitylation on the same protein. PR619 treatment leads to inhibition of platelet spreading on glass, 11 consistent with a role for ubiquitin turnover in the regulation of outside-in signalling through the αIIbβ3 integrin, as well as in CRP-XL-induced integrin activation ( Fig. 1E ).

### Ubiquitin Pathway

Several diGly-tagged peptides from ubiquitin were detected, consistent with platelets containing a range of polyubiquitin chains with K6, K11, K27, K48 and K63 linkages ( Fig. 4D ). An increase in peptide abundance of more than twofold was observed for all of these. K48-linkages target proteins to the proteasome, whereas others, such as K63, have a role in signalling. 4 Some proteasome sub-units (ADRM1, PSMA1, PSMC1, 3 and 6, PSMD1, 3) also showed ubiquitylation ( Supplementary Table S1 , available in the online version). Ubiquitylation of ligases can be a sign that they are active and tags on several E2, E3 ligases and deubiquitinating enzymes are apparent, with several showing increases on CRP-XL treatment.

Protein modification by ligation of the ubiquitin-related protein Nedd8 also leads to a diGly-tagged residue following trypsin cleavage. The only well-characterized targets for Nedd8 ligation are cullins 38 and several cullins are found in the human platelet proteome. 21 We detected diGly-tags on cullin-2, cullin-5 and cullin-9, as well as the Nedd8 E1 activating enzyme UBA3, and Nedd8 conjugating enzymes Ubc12 (UBE2M) and UBE2F ( Supplementary Table S1 , available in the online version). DiGly-tags were also detected on Nedd8 itself consistent with at least five different linkages, three of which changed by more than twofold on CRP-XL treatment.

### Cytoskeleton and Vesicle Trafficking Components

Consistent with a previous report of ubiquitylation of both talin and filamin, 10 we detected 65 different peptides containing diGly-tags derived from talin, 39 sites changing more than twofold on stimulation, all but one increasing, and 35 diGly-tagged peptides derived from filamin, 20 increasing by at least twofold ( Supplementary Table S1 , available in the online version). MS analysis also revealed extensive ubiquitylation for myosin-9, with 40 diGly-tagged peptides, 30 of which significantly increased following stimulation. Thirteen members of the Rab family of small G proteins, involved in vesicle trafficking, and other small G proteins such as Rac1 and Ral1 are also ubiquitylated, some of which have been implicated in platelet function. 39 40 41 42 Kinases regulated by monomeric G proteins also showed altered ubiquitylation: ROCK2 decreases whereas CDC42 kinase increases ( Supplementary Table S3 [available in the online version], Fig. 2C ).

### Identification of Ubiquitin-Containing Complexes by TUBEs Enrichment

**Fig. 5:**
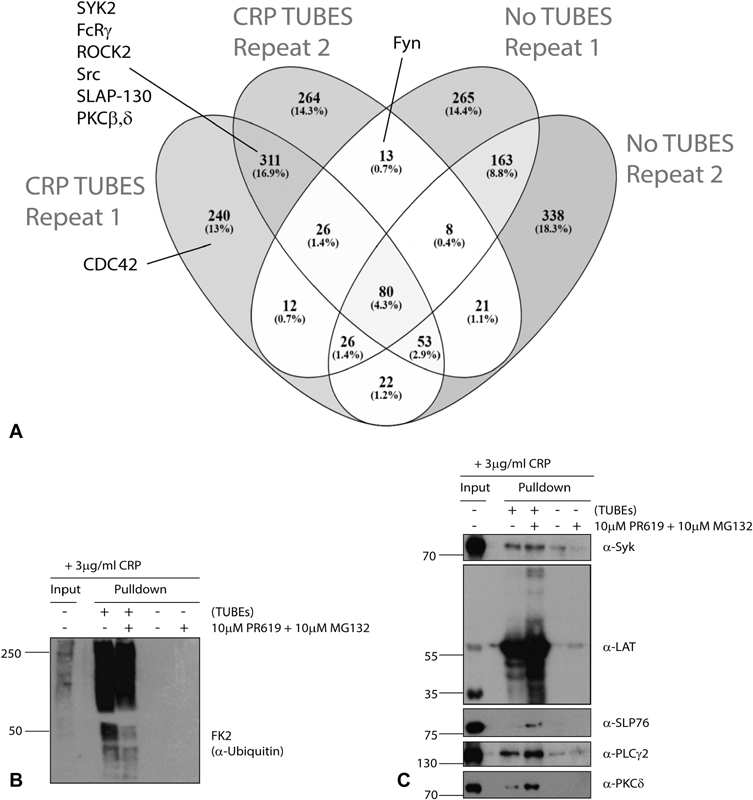
Enrichment of complexes containing ubiquitin from collagen-related peptide (CRP-XL) stimulated human platelets using GST-Tandem Ubiquitin Binding Entities (GST-TUBEs). ( **A** ) Venn diagram showing overlap of proteins identified by mass spectrometry in TUBE-based pull-down experiments to enrich for poly-ubiquitylated material in platelets 5 minutes after activation with CRP-XL (3 μg/mL). Proteins were enriched by glutathione sepharose chromatography following incubation of lysates with GST-TUBEs or GST alone and identified by mass spectrometry. ( **B** ) Human platelets were stimulated with CRP-XL (3 μg/mL) for 5 minutes in the presence of the proteasome inhibitor MG132 (10 μM) and the pan-deubiquitylase inhibitor PR619 (10 μM). Whole cell lysates were enriched for ubiquitin by association with GST-TUBEs and enrichment confirmed by Western blot using FK2 (left panel). Samples were probed using antisera specific for Syk, LAT, SLP76, PLCγ2 and PKCδ signalling proteins (right panel).

In a separate analysis, protein complexes containing ubiquitin were pulled down from CRP-XL-stimulated platelets by incubating extracts with GST-tagged TUBEs or GST alone as a control. TUBEs contain tandem ubiquitin binding domains which increase their affinity for ubiquitylated proteins. 34 The resulting eluates were analysed by MS ( Supplementary Table S4 , available in the online version) and the overlap in proteins between two repeats is shown in Fig. 5A . Ubiquitin was present in the samples pulled down by TUBEs, but not in the absence of TUBEs, verifying enrichment ( Fig. 5B ). A total of 1,076 proteins were identified in the eluates only in the presence of TUBEs with the highest scores for filamin, talin and myosin-9, consistent with the multiple diGly peptides identified from these proteins. A total of 815 of these proteins were not identified in the absence of TUBEs, and in this control, the protein with the highest score was GST itself. Several other proteins identified in the diGly immunoprecipitates were also selectively identified in the TUBEs pull-downs, including pleckstrin, protein kinase C, integrin-linked kinase (ILK) and Syk. Several platelet receptors such as GPIb, GPIX, GPV and GPVI were present in the TUBEs pull-downs. GST-TUBEs enrichment followed by Western blot analysis confirmed the enrichment of several proteins identified in either the diGly-peptide or TUBEs MS analysis ( Fig. 5B ). In the case of LAT, higher molecular weight forms were enriched in the bound fraction, consistent with direct polyubiquitylation.

## Discussion

Platelet aggregation and dense granule secretion triggered by CRP-XL are inhibited by proteasome and pan-deubiquitylase inhibitors, demonstrating a role for ubiquitylation downstream of GPVI. This is consistent with previous reports of activating platelets with collagen, which works through both GPVI and integrin signalling, 8 10 11 and that platelets from mice deficient in the ubiquitin ligase c-Cbl are hyperactivated in response to GPVI agonists, correlating with loss of Syk ubiquitylation, 12 13 suggesting a specific role for ubiquitylation in the GPVI signalling pathway.

This work identifies a large number of proteins modified by ubiquitylation in resting human platelets and characterizes changes on stimulation of the GPVI signalling pathway by CRP-XL. Platelets were analysed in the presence of indomethacin and apyrase to prevent any potential changes in ubiquitylation caused by secondary mediators such as adenosine triphosphate and thromboxane. The direct effect of these secondary mediators on ubiquitylation is unknown. The deubiquitylase inhibitor PR619 also inhibits activation of the platelet integrin αIIbβ3 as measured by fibrinogen binding, suggesting it works, at least in part, directly on the GPVI-induced signalling pathway, upstream of integrin activation. Fragmentation of proteins with trypsin and subsequent immunoprecipitation with antisera specific for the diGly-tag indicative of ubiquitylation reveals extensive ubiquitylation of proteins in resting platelets and changes, mainly increases but some decreases, following activation of platelets by CRP-XL. Increase in abundance of peptides could represent increased activity of ubiquitin ligases, decreased activity of deubiquitylases or decreased degradation, whereas a decreased abundance could indicate activation of deubiquitylases, inhibition of ligases or increased degradation. In an alternative approach, in CRP-XL-stimulated extracts, complexes containing ubiquitylated proteins were pulled down using GST-tagged TUBEs and eluted proteins identified by MS. Complete overlap between the diGly and TUBEs datasets was not expected as the TUBEs analysis enriches for complexes containing ubiquitylated proteins but not all complex components are ubiquitylated. For example, the TUBEs pull-downs (R2) but not the diGly peptide immunoprecipitates contained GPVI which forms a complex with FcRγ-chain which in turn has four direct sites of ubiquitylation. Conversely, TUBEs will not enrich for proteins whose ubiquitin modifications are masked by binding proteins in a complex, whereas the diGly immunoprecipitation involves free peptides and so all sites should be exposed. The combination of complimentary approaches is presented to generate the most extensive database and the large numbers of proteins identified in these analyses confirm the extensive use of this modification in human platelets.

**Fig. 6:**
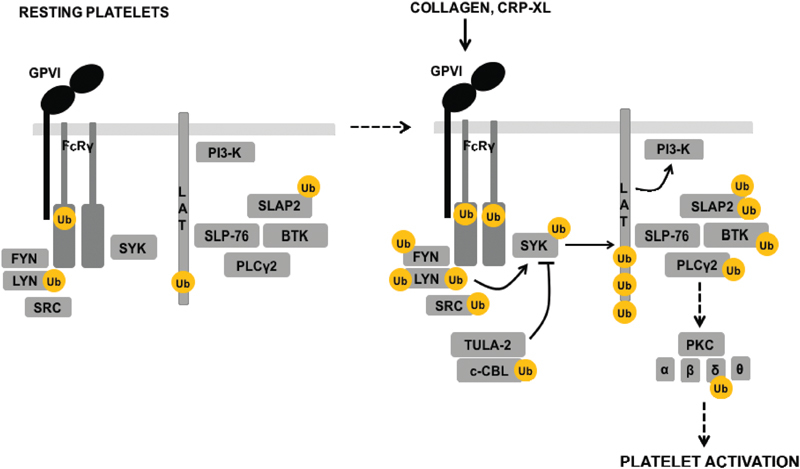
Schematic of the ubiquitylation of glycoprotein (GP) VI signalling pathway. The major players in the GPVI signalling pathway and their ubiquitylation status before and after stimulation.

There is widespread ubiquitylation of proteins involved in the GPVI signalling pathway ( Fig. 6 ), some on multiple sites, with simultaneous modification of two closely adjacent sites on Syk, Lyn and FcRγ chain. The function of multiple sites of ubiquitylation, such as 17 in Syk, is not known. In B cells, the aminopeptidase DPP9 cleaves the two N-terminal amino acids of Syk following activation of the B cell receptor, thus generating a substrate for the N-end rule degradation pathway by UBR1/3 E3 ligases, and inhibition of DPP9 leads to Syk stabilization. 43 Platelets also express DPP9, 21 so a similar pathway in operation in platelets could explain the extensive number of ubiquitylation sites and raises the possibility of a similar degradation pathway for Lyn. Several of the ubiquitylation sites lie in the vicinity of known phosphorylation sites. For Syk, platelet activation by CRP-XL in the presence of PR619, but not MG132, led to an increase in tyrosine phosphorylation. This correlates with increased ubiquitylation and is consistent with previous reports that enriching for ubiquitylated Syk also enriches for active Syk phosphorylated on Y525/526. 12 In PLCγ2, the single ubiquitylation site identified (K862) is close to two known phosphorylation sites (T857 and Y858). All three sites of ubiquitylation in the integrin β3 subunit lie in the C terminal cytosolic tail which forms a platform for interaction with downstream signalling proteins including talin, Src, Syk and ILK (reviewed by Durrant et al 44 ), raising the possibility that ubiquitylation may influence these interactions.

Several proteins whose function in platelets is not well characterized nevertheless show extensive ubiquitylation. For example, 29 diGly-tagged peptides were identified from serum deprivation-response protein, 12 of which changed on stimulation. This protein interacts with pleckstrin 45 and is also extensively phosphorylated, 36 suggestive of a regulatory role in platelet function. Interestingly, two sites of ubiquitylation are consistently detected in β amyloid protein. The precursor of this modulates venous thromboembolism in mice 46 and platelets have been hypothesized to be a source of β amyloid protein in plaques in brains of Alzheimer's patients. 47 Inhibition of β amyloid degradation has been implicated in plaque formation, 48 so it would be of interest to know if its ubiquitylation in platelets leads to degradation and if disruption of this contributes to disease progression.

The extent and diversity of the ubiquitome is comparable to that of the platelet phosphoproteome analysed following ADP stimulation, 36 and targeting this pathway has the potential to modulate platelet functional responses. It has already been demonstrated that small molecules that inhibit the proteasome or deubiquitylases inhibit functional platelet activation 8 9 10 11 and that platelets isolated from mice deficient in the ubiquitin ligase c-Cbl are more sensitive to activators of GPVI than platelets from wild-type mice. 12 13 The comprehensive mapping of the ubiquitome is a first step towards understanding the complexity and mechanisms of ubiquitylation in platelet biology. An increasing number of reagents which more specifically target individual members of the deubiquitylase or ubiquitin ligase family are being developed, 49 50 raising the possibility that these could act as specific modulators of platelet functional responses.

## Notes

**What is known about this topic?**

- Proteasome and deubiquitylation inhibitors block human platelet aggregation suggesting a critical role for ubiquitylation in platelet activation.
- Ubiquitylation of the tyrosine kinase Syk is ubiquitylated in response to activation of GPVI by collagen and has been proposed to mediate down-regulation of platelet activation.
- Talin and filamin also are ubiquitylated in human platelets.

**What does this paper add?**

- Resting platelets contain significant levels of ubiquitylated proteins. In this study, we identified 1,116 ubiquitylated peptides derived from 476 proteins.
- Activation of GPVI leads to extensive remodelling of the ubiquitome, with 925 ubiquitylated peptides changing more than twofold. In total, 1,634 ubiquitylated peptides derived from 691 proteins were detected.
- Syk contains at least 17 sites of ubiquitylation, all increasing upon activation of GPVI.
- Inhibition of deubiquitylation inhibits GPVI-dependent platelet aggregation and secretion.
- Small molecule reagents which specifically target individual members of the deubiquitylase or ubiquitin ligase have therapeutic potential as specific modulators of platelet functional responses.

## Supplementary Material

Supplementary Table Captions Supplementary Table Captions
