# Supplementary material for: Human Platelet Protein Ubiquitylation and Changes following GPVI Activation
Source: Thromb Haemost. 2018 Dec 31;119(1):104–16. doi: 10.1055/s-0038-1676344 (PMC6327716; doi:10.1055/s-0038-1676344)
Supplement: Supplementary file 1 — Supplementary Table Captions [file 10-1055-s-0038-1676344-s180396.doc]

**Supplementary Information**

**Supplementary Tables**

**Supplementary Table S1** Platelet protein ubiquitylation sites (diGly) identified by tandem mass spectrometry—all proteins. Protein identification was performed using Mascot, and label-free quantitation conducted using the Progenesis IQ software. The columns include Accession: UniProt accession number and gene name; Description: protein name; Score: peptide probability-based identification score; Sequence: peptide sequence; Modifications: type of modifications with amino acid position in (); Max fold change: abundance ratio; Highest: group with the highest abundance value; Lowest: group with the lowest abundance value; ANOVA: analysis of variance (ANOVA) for change in abundance (fold change, *p* < 0.05); Maximum CV: maximum coefficient of variation (%); Peptide identifier: retention time _ mass; Ions used in quantitation: number of ions; Ions: number of precursor mass ions matched to this peptide sequence; De-convoluted peptide ions: number of MS/MS query/queries; De-convoluted charges: ion charge used for precursor ion selection; Retention time (min): peak retention time observed in liquid chromatography (LC); Neutral mass: peptide monoisotopic mass (calculated); Normalized abundance: ion intensities, NS – not stimulated, Stim – CRP stimulated (two technical repeats); Raw abundance: raw ion intensities, NS – not stimulated, Stim – CRP stimulated (two technical repeats); Spectral counts: number of MS/MS spectra, NS – not stimulated, Stim – CRP stimulated (two technical repeats); Experiment number: R1 – experiment 1 (green), R2 – experiment 2 (red), R3 experiment 3 (blue).

**Supplementary Table S2** Platelet protein ubiquitylation sites (diGly) identified by tandem mass spectrometry—*p* < 0.05 fold change. From Supplementary Table S1, all proteins with a significant fold-change significance (*p* < 0.05) are shown. For the description of content shown in each column, please see legend to Supplementary Table S1.

**Supplementary Table S3** Platelet protein ubiquitylation sites (diGly) identified by tandem mass spectrometry—*p* < 0.05 fold change/> twofold. Sub-set of proteins listed in Supplementary Table S1, for which a more than twofold change with *p* < 0.05 analysis of variance (ANOVA) significance was observed. For the description of content shown in each column, please see legend to Supplementary Table S1.

**Supplementary Table S4** Collagen-related peptide (CRP)-induced ubiquitylated proteins identified by Tandem Ubiquitin Binding Entity (TUBE) pull-down and mass spectrometry analysis. Proteins identified by tandem mass spectrometry that are shared between TUBEs pull-down experiments (two technical replicates – A:+CRP_TUBES(R1); B:+CRP_TUBES(R2)), but not present in no-TUBE pull-down control experiments using CRP-stimulated samples (two technical replicates – C:+CRP_noTUBES(R1); D:+CRP_noTUBES(R2)) are shown (UniProt accession number), providing the basis for the Venn diagram shown in Fig. 5A. Data of one representative out of three independent biological experiments is shown.
